# Using Biplanar Fluoroscopy to Guide Radiopaque Vascular Injections: A New Method for Vascular Imaging

**DOI:** 10.1371/journal.pone.0097940

**Published:** 2014-05-16

**Authors:** Haley D. O’Brien, Susan H. Williams

**Affiliations:** 1 Ohio University, Department of Biological Sciences, Graduate Program in Ecology and Evolutionary Biology, Athens, Ohio, United States of America; 2 Ohio University Heritage College of Osteopathic Medicine, Department of Biomedical Sciences, Athens, Ohio, United States of America; Medical University Innsbruck, Austria

## Abstract

Studying vascular anatomy, especially in the context of relationships with hard tissues, is of great interest to biologists. Vascular studies have provided significant insight into physiology, function, phylogenetic relationships, and evolutionary patterns. Injection of resin or latex into the vascular system has been a standard technique for decades. There has been a recent surge in popularity of more modern methods, especially radiopaque latex vascular injection followed by CT scanning and digital “dissection.” This technique best displays both blood vessels and bone, and allows injections to be performed on cadaveric specimens. Vascular injection is risky, however, because it is not a standardizable technique, as each specimen is variable with regard to injection pressure and timing. Moreover, it is not possible to view the perfusion of injection medium throughout the vascular system of interest. Both data and rare specimens can therefore be lost due to poor or excessive perfusion. Here, we use biplanar video fluoroscopy as a technique to guide craniovascular radiopaque latex injection. Cadaveric domestic pigs (*Sus scrofa domestica*) and white-tailed deer (*Odocoileus virginianus*) were injected with radiopaque latex under guidance of fluoroscopy. This method was found to enable adjustments, in real-time, to the rate, location, and pressure at which latex is injected in order to avoid data and specimen loss. In addition to visualizing the injection process, this technique can be used to determine flow patterns, and has facilitated the development of consistent markers for complete perfusion.

## Introduction

Studies of arterial or venous morphology have yielded much insight into the physiology, function, and evolution of many vertebrate taxa. For example, Georgian era observations of dense arterial retia in the forelimbs of slow-moving mammals like lorises (*Loris tardigradus*) and three-toed sloths (*Bradypus tridactylus*) [1], led to the functional hypothesis that such morphology is a requisite for a strong, prolonged prehensile grip [2]. More recently, models of cranial vasculature have been used to explain why giraffes (*Giraffa camelopardalis*) do not faint from blood pressure changes during head-raising [3]. Vascular studies have also revealed the morphology underlying complex and unique physiologies, such as deep-water, dive-induced nitrogen absorption in the bottlenose dolphin (*Tursiops truncates*) [4], sophisticated selective brain cooling of artiodactyls [5] and birds [6], and lingual pump feeding in flamingos (*Phoenicopterus ruber*) [7]. Evolutionary processes and phylogenetic relationships within eutheria [8], [9], [10], primates [11], and cetaceans [12] have also been gleaned from vascular studies. Although the study of vascular morphology is an integral part of research in zoology, historical data collection methods are generally destructive and frequently unreliable.

The most traditional methods for vascular data collection, gross dissection and serial sectioning, have existed for hundreds of years (see e.g. The Edwin Smith Papyrus, 1500 BCE, [13]; the *Hippocratic Corpus*, 4^th^ century BCE, [14]) and have provided anatomists with information regarding the three-dimensional relationships of distributing vessels to the vascular tree and to other structures [15], [16]. These destructive methods occasionally lead to inaccurate descriptions documented in the literature by discrepancies regarding the anatomy of even large arteries. For example, dromedary camels (*Camelus dromedarius*) are reported to either lack an internal carotid artery [17], possess a rudimentary internal carotid artery [18], or to have an internal carotid artery of large size [19]. Moreover, the anatomy of vessels passing through or behind hard tissues is difficult to capture [19]. Resin and corrosion casting have addressed some of these issues, enabling studies of the interaction between blood vessel and bone without the obfuscation of other soft tissues [20], [21]. These are, however, inherently destructive techniques that fail to fully resolve anatomical relationships of vasculature in relation to the surrounding hard and soft tissue structures.

More recent techniques, such as those employing radiography, aim to increase the accuracy with which vasculature and surrounding structures can be documented. Radiography-based studies involve injection of viscous, radiopaque contrast agents (e.g. barium sulfate-infused latex) into large blood vessels, followed by x-ray [22], [23], [24], [25], stereoangiography [26], or computed tomography (CT) scan [7] imaging techniques. Performing these non-destructive techniques prior to standard destructive methodologies has several key advantages. First and foremost, the relationships of hard and soft tissues are maintained. This is conducive to discovery of novel vascular structures such as the paralingual sinuses in flamingoes (*Phoenicopterus ruber*) [7] and dense vascularization of the melon region of bottlenose dolphins (*Tursiops truncates*) [4]. Additionally, osteological correlates–consistent patterns of hard and soft tissue interaction–can be documented, even as arteries pass through bone and within bony canals. Identification of these correlates is highly useful in paleobiology, wherein hard tissues must be used to draw inferences regarding soft tissue morphology and function [27], [28].

**Figure 1:**
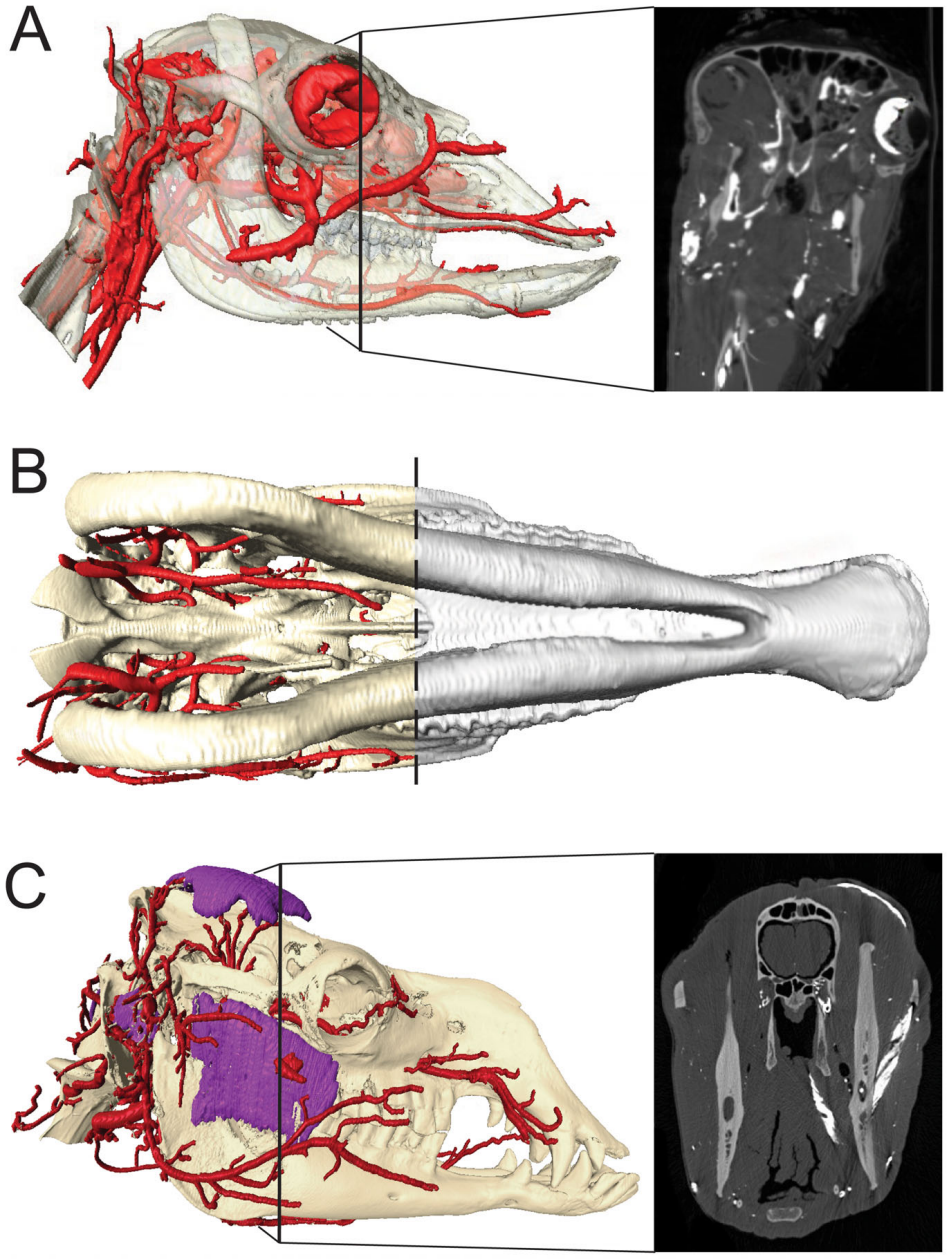
Specimens injected without fluoroscopy. Each of these specimens exemplifies data loss from blind-process perfusion. A) Lateral view; over-perfusion of latex in an arterial study of *Vicugna pacos* (OUVC), with abundant latex in the eyeball and venous system. B) Ventral view; under-perfusion (gray) in *Equus ferus caballus* (OUVC). Arteries are not perfused rostral to the sphenoid bone. C) Lateral view; overflow of latex into the fascial sheaths surrounding the muscles of mastication in *Camelus dromedarius* (AMNH M-200462).

The main detractor from radiographic studies of vasculature is that these injections are essentially a blind process. The inability to monitor injection and perfusion of contrast agents can result in incomplete perfusion, over-perfusion (“blow-outs”), and/or back-flow through veins (Figure 1). Here we propose a novel method that enables visualization of the injection process in real-time using biplanar videofluoroscopy. We implemented this method with the goals of determining the extent of injection medium perfusion in real-time and identifying consistently reliable markers of perfusion in order to better standardize non-fluoroscopic injection procedures.

## Materials and Methods

Three white tailed deer (*Odocoileus virginianus*) heads were obtained as refuse from Woodroad Smokehouse and Game Processing in Southeastern Ohio. Specimens were immediately transported to Ohio University and stored frozen until the time of study. Additionally, heads of two domestic pigs (*Sus scrofa domestica*) were obtained as refuse from an in-house study outside the scope of this research, and were also stored frozen until the time of study. Prior to utilization, all specimens were inspected for putrefaction and any damage that may have been caused by freezing. For comparison only, several specimens were injected using a similar protocol, but without the guidance of fluoroscopy, and using traditional indicators of perfusion (e.g. appearance of brightly colored latex in mucosal arterioles). These include similarly-preserved specimens of *Vicugna pacos* (Ohio University Vertebrate Collections [OUVC]-SHW1) and *Equus ferus caballus* (OUVC-SHW2), as well as an alcohol-preserved specimen of *Camelus dromedarius* on loan from the American Museum of Natural History (AMNH M-200462). Permissions were obtained to carry out this study using specimens loaned from the AMNH, OUVC, and donated cadaveric specimens. No animals were sacrificed for the purpose of this study. In this context, compliance with the guidelines for care and use of laboratory animals is not required.

Specimen preparation followed Holliday et al. [7]. After specimens were thoroughly thawed, the common carotid arteries were isolated in dissection. An 18-gauge angiographic cannula (BD Medical Angiocath Autoguard IV Shielded) was inserted into either the right or left common carotid, and secured with surgical ligature and adhesive. Cannulae were connected to 30 mL veterinary luer-lok needleless syringes (BD Medical) via 10 ft. of 0.125 in. external diameter clear PVC tubing (Thermo Scientific). The length of tubing reflects the need to perform injections from behind a leaded glass barrier, in compliance with Ohio University radiation safety protocols. To maintain clarity of imaging in this study, the venous system was not injected. It should be noted, however, that this method is particularly useful for the more compliant, valve-ridden venous system. The arterial system was flushed with water for 10 minutes, followed by perfusion with 90 mL of 10% One-Point anticoagulant solution. Continued preparation for fluoroscopy deviates from the methods of Holliday et al. [7], as large, severed distributing vessels on the dissected surface of the neck (e.g. vertebral arteries, thyroid arteries, and muscular branches of the external carotid and occipital arteries) must be ligated to prevent excessive discharge of contrast agent during the somewhat remote injection process.

**Figure 2:**
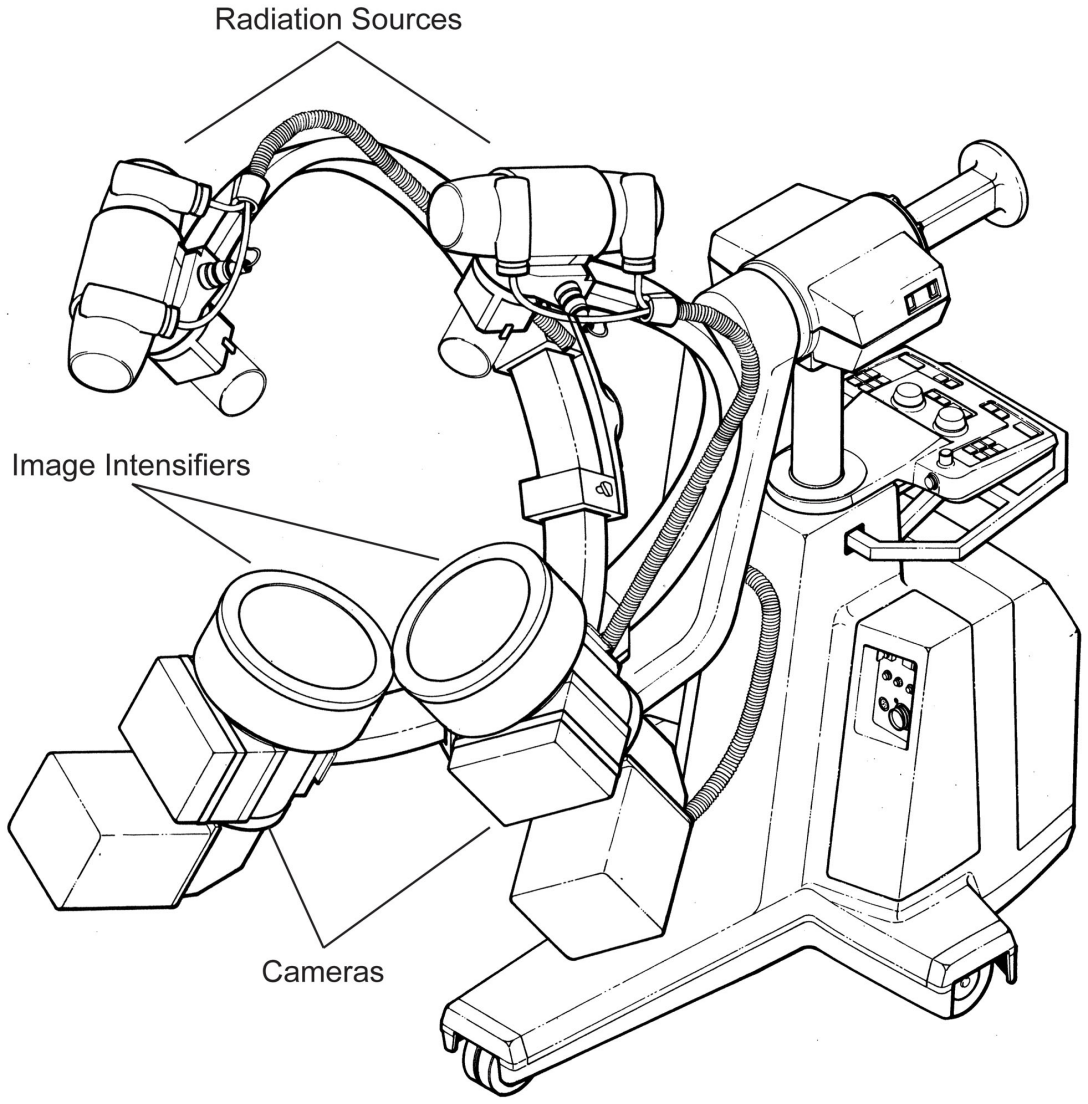
Fluoroscopes. Schematic of synchronized fluoroscopes (OEC-9000) retrofitted with 30 cm image intensifiers and Qualysis Oqus 310 cameras.

**Figure 3:**
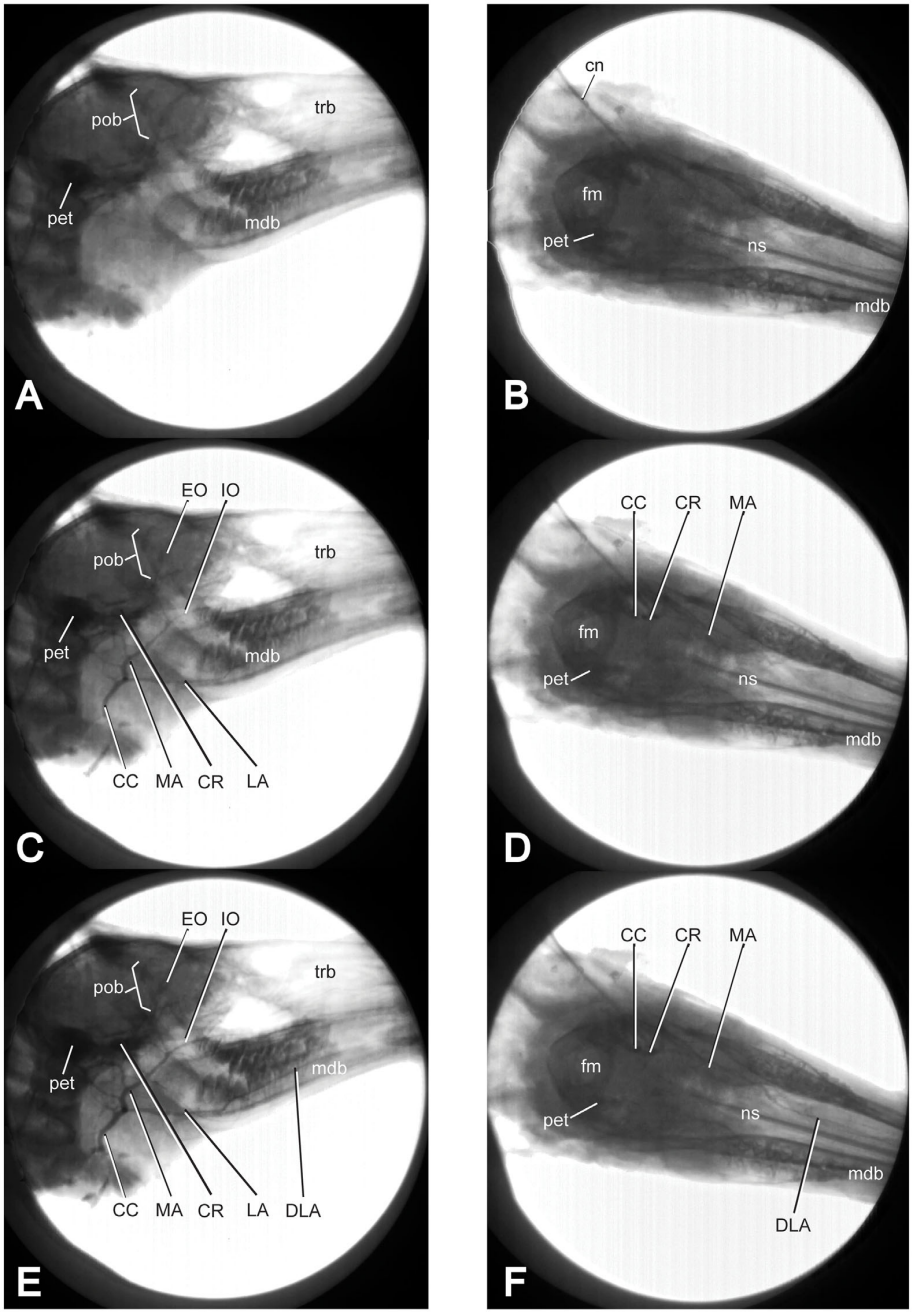
Real-Time Fluoroscopic Injection. Screen captures taken during video fluoroscopically-guided injection of *Odocoileus virginianus* (OUVC). A) Right lateral view and B) Dorsal view prior to injection. C) Right lateral view and D) Dorsal view 13 seconds into perfusion; E) Right lateral view and F) Dorsal view 38 seconds into perfusion. Dense bone and radiopaque latex appear black; soft tissues and laminar bones (e.g. turbinates) are gray scale. Abbreviations: CC, common carotid artery; CR, carotid rete; DLA, deep lingual artery; EC, external carotid; EO, external ophthalmic artery; fm, foramen magnum; IO, infraorbital artery; LA, lingual artery; MA, maxillary artery; mdb, mandible; ns, nasal septum; pet, petrosal; pob, postorbital bar; trb, turbinates.

Fluoroscope set-up follows that typical of X-ray Reconstruction of Moving Morphology methodology, or XROMM [29], [30]. Two synchronized fluoroscopes (OEC-9000) retrofitted with 30 cm diameter image intensifiers (Figure 2) were used to monitor the injection process. Prior to perfusion, specimens were staged on the image intensifiers so that radiographic digital video could be recorded for dorsal and lateral aspects of the cranium simultaneously (Figure 3). Videos of the injection were recorded at 20 frames per second using a Qualysis motion capture system consisting of two Oqus 310 cameras. One camera was mounted to the output port on the image intensifiers of each fluoroscope. Because all injections took place behind a leaded glass barrier, the external surface of the specimens was monitored for latex extravasation using a closed-circuit webcam synchronized with the fluoroscopy cameras. Fluoroscope intensity was calibrated using a pre-injected white-tailed deer, and set to 70–90 kVp and 3.5–4.0 mA, depending on the specimen, for recording.

Specimens were injected with a solution of 40% Liquid Polibar Plus barium sulfate suspension (BaSO_4_, E–Z-Em, Westbury, NY) in red liquid latex injection medium (Ward’s, Rochester, NY) [26]. This ratio of radiopaque barium to latex yields ideal contrast for imaging [26], and perfused vessels were visible throughout the injection process. Injection was performed manually, without the use of injection reservoirs or pumps, using intermittent pulses of pressure and under real-time fluoroscopic visualization. This method ensured that perfusion could be ceased if or when potential problems arose. Mitigation of perfusion problems included adjustment of rate and pressure of injection, as well as relocation of the cannula into alternative arteries (e.g. facial artery). The volume injected varied per specimen (12 to 22 mL), with perfusion continuing until the rostral-most distributing arteries were radiopaque and latex emerged from the contralateral (non-cannulated) common carotid artery. Acetic acid soaked cotton (10% glacial acetic acid solution) was used to prevent leaks, as acetic acid immediately sets latex.

### Analysis

**Figure 4:**
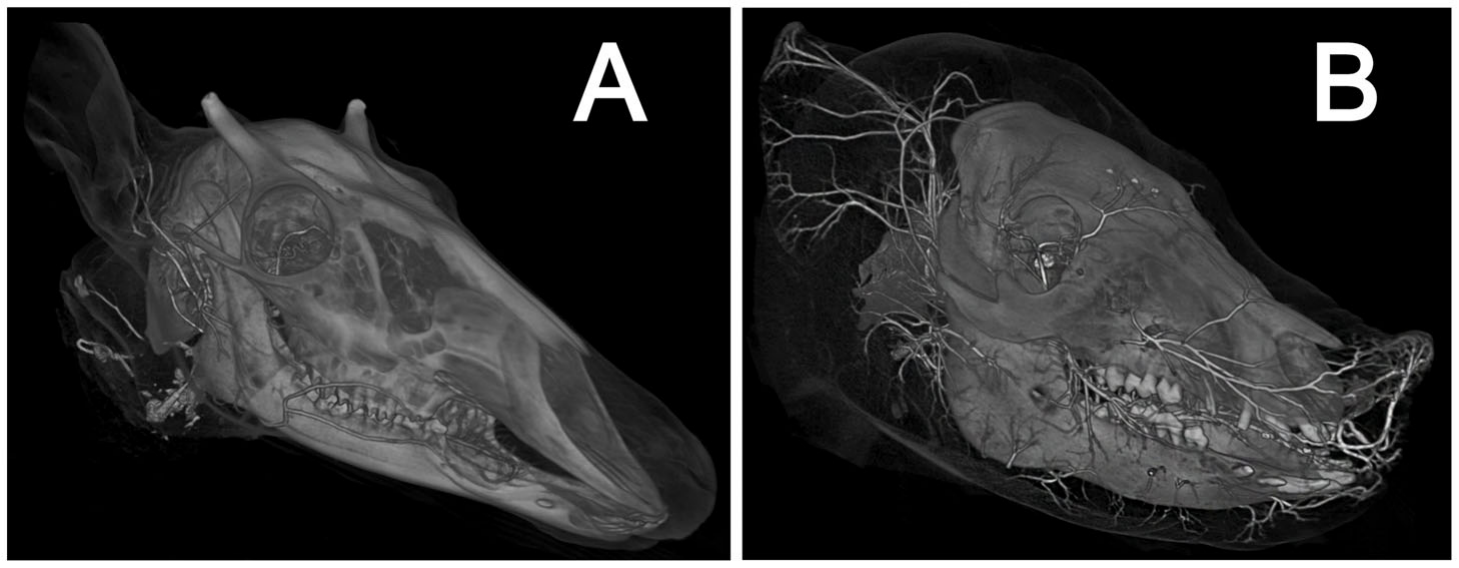
Degree of perfusion achieved. Volume rendering of A) *Odocoileus virginianus* (OUVC) and B) *Sus scrofa domestica* (OUVC), demonstrating degree of perfusion. In both specimens, small arterioles are perfused into the rostral most portion of the rostrum (especially apparent in *Sus*), and the distal-most portion of the pinna. In a volume rendering, opacity is density dependent, with the highest density voxels (in this case, barium-perfused arteries and ossified structures) projected as the most opaque.

**Figure 5:**
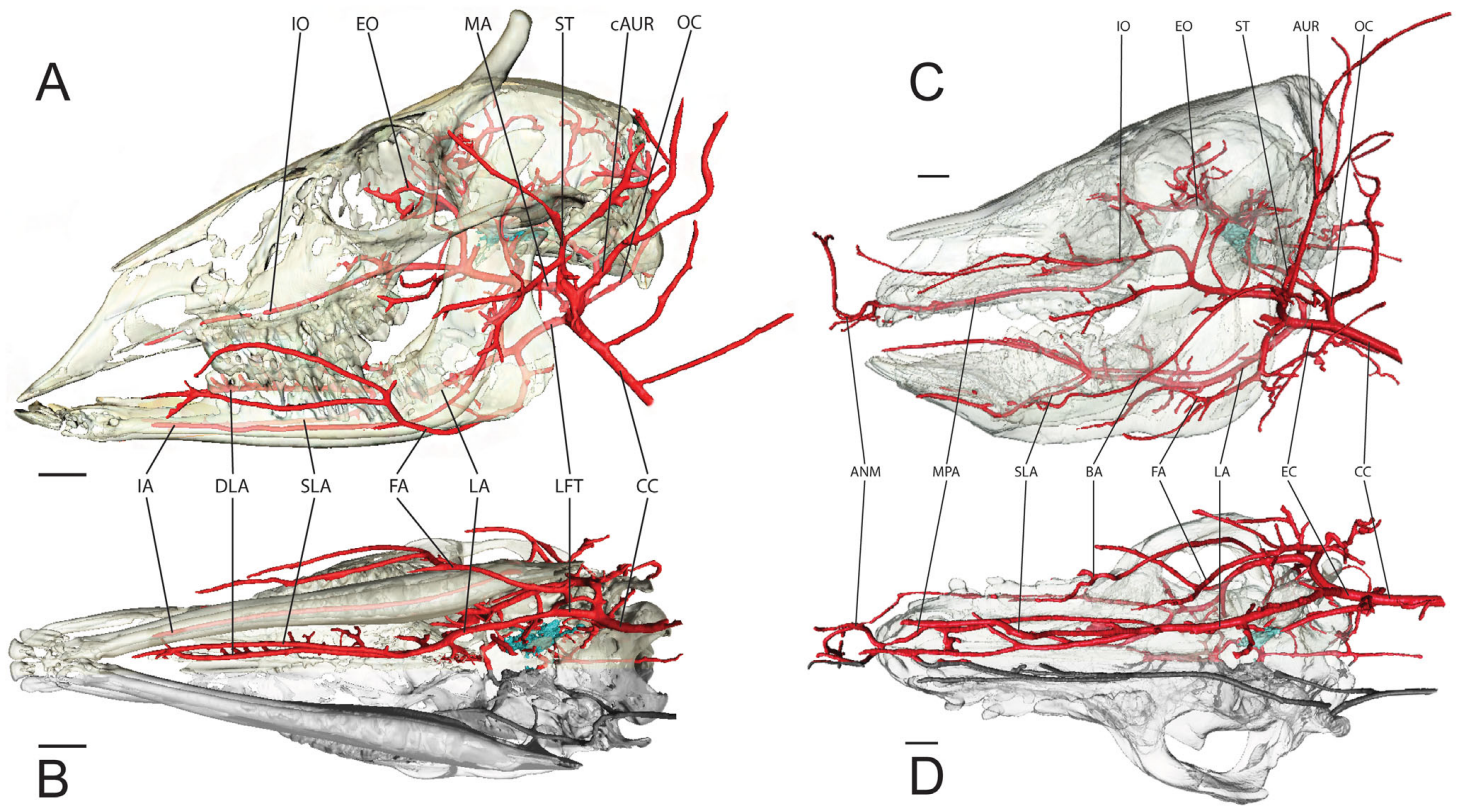
Simplified Arterial Models. Stream-lined surface segmentation of the cranial arteries (red), carotid rete (turquoise) and osteology (semitransparent) of: the White-Tailed Deer, *Odocoileus virginianus* (OUVC) using fluoroscopy-guided vascular injection, CT imaging, and segmentation analysis. A) Left lateral view. B) Ventral view. Scale bar = 2 cm. During injection, fluoroscope 1 was set to 80 kVp and 3.5 mA; fluoroscope 2 was set to 70 kVP and 3.5 mA); and of *Sus scrofa domestica* (OUVC) in C) Left lateral view and D) Ventral view. Scale bare = 2 cm. For this injection, fluoroscope 1 was set to 87 kVP and 3.8 mA; fluoroscope 2 was set to 90 kVp and 4.0mA. The mandible has been digitally removed for clarity. Abbreviations: ANM, artery of nasal mucosa; AUR, auricular arteries; cAUR, caudal auricular artery; BA, basilar artery; CC, common carotid artery; CR, carotid rete; DLA, deep lingual artery; EC, external carotid; EO, external ophthalmic artery; FA, facial artery; IA, inferior alveolar artery; IO, infraorbital artery; LA, lingual artery; LFT, linguofacial trunk; MA, maxillary artery; MPA, major palatine artery; ns, nasal septum; OC, occipital artery; SLA, sublingual artery; ST, superficial temporal artery.

Qualisys Track Manager (version 2.8; Qualisys Motion Capture Systems) video analysis software was used to track flow of latex and determine perfusion patterns. After injection, all specimens were CT scanned at the Holzer Clinic in Athens, Ohio, on a Philips Brilliance 64 slice CT scanner, at 0.67 mm slice thickness, 150 kV, 80 mA, and yielding a voxel size of 0.693359×0.693359×0.5 mm. The resulting data were analyzed in Avizo (version 7.0; VSG). To ease handling of the data, all specimens were cropped to eliminate unusable data (e.g. air, packing material), and digitally bisected on the side of cannulation. Scan data were then up-sampled to yield a voxel size of 0.1×0.1×0.1 mm. Up-sampling does not affect the inherent quality of the data, and was implemented to improve 3-D models of lower-resolution CT scans of these large specimens by artificially increasing pixel size and resulting in a visually smoother surface. Degree of perfusion was assessed using volume rendering (Figure 4), a technique that visualizes voxels based on their intensity (a proxy for material density), projecting the highest intensity as more opaque, and lower intensities as more transparent. The skull and arteries were then segmented, first by isolating distinctive gray-scale values, and then manually inspected and edited to verify accuracy. To aid identification of distributing arteries, stream-lined versions of the segmented morphology were then rendered in 3-D (Figure 5).

## Results

### Perfusion

Benefits of fluoroscopy-guided injection included adjustment of the perfusion process in response to potential errors, complete perfusion, identification of reliable indicators of perfusion, and documentation of flow patterns. The highly viscous latex-barium solution is incapable of perfusion into capillary beds [7] so “complete” perfusion is defined as perfusion into the terminal arterioles of all large divisions of cranial arteries. Under these conditions, complete perfusion was achieved in all five specimens using fluoroscopy (Figure 5). In contrast, perfusion was flawed in all three specimens that followed a blind injection protocol (Figure 1). Use of fluoroscopy enabled identification of reliable perfusion markers. The most consistent *internal* indicator of complete injection was perfusion of barium-infused latex into the rostral-most arteries (e.g. nasal and labial arteries). Reliable *external* markers of complete perfusion were also identified. The most consistent external marker is back-flow through the contralateral common carotid artery, which always occurred after complete rostral perfusion. Back-flow through the vertebral arteries was found to be a poor indicator of perfusion, as latex injected into the common carotid artery almost immediately reaches the vertebral artery and its extensive system of anastomoses. Back-flow through muscular arterial branches on the injection side is also a poor indicator of perfusion. From extensive experience with vascular injection, continuing injection until contralateral muscular arterial branches results in a higher percentage of perfusion on both sides of the cranium, but at the risk of blowouts (e.g. Figure 1A). Additional external markers can include appearance of brightly-colored latex in small vessels of mucosal tissues (oral surface of the lip; inner eyelid), however, these are unhelpful in animals with heavily pigmented mucosae and reliance on these indicators can lead to over-perfusion.

### Perfusion Patterns

Tracking the flow of the radiopaque latex assisted in identification of better indicators of perfusion. In *Sus scrofa domestica,* the lengthy common carotid artery bifurcates posterior to the mandibular ramus into a homologous internal carotid artery and an external carotid artery. Injection medium flows from this bifurcation almost directly into the carotid rete and subsequent cerebral arteries. Immediately following the bifurcation of the internal and external carotid arteries, latex flows concomitantly into pharyngeal, infra- and supra-hyoid, temporal, occipital, linguofacial, and maxillary branches. From the maxillary artery, perfusion into the rostral rami ad rete mirabile caroticum occurs, followed by perfusion into the cerebral arterial circle (of Willis) and the ophthalmic arteries. Finally, upon the injection medium reaching the rostral-most extent of these distributing arteries and their larger tributaries, retrograde flow through the vertebral and non-cannulated common carotids indicates that perfusion is complete. See figures S1 and S2: Video recording of fluoroscopy-guided radiopaque latex arterial injection of *Sus scrofa domestica* in (Figure S1) lateral view, and (Figure S2) dorsal view.

In *Odocoileus*, injection medium ascends from the common carotid artery into the large maxillary artery and the smaller linguofacial trunk. As the maxillary artery fills with injection medium, arterial branches to the infra- and supra-hyoid musculature begin to be perfused, as do occipital and temporal branches. The maxillary tributaries are essentially perfused in unison. This includes filling of the caudal rami of the carotid rete, followed shortly thereafter by the rostral rami. These rami fill the carotid rete in tandem from the anterior and posterior aspects. From this point, perfusion continues throughout the Circle of Willis and the remainder of the distributing arteries of the cranium. Once injection medium has reached the rostral-most extent of these arteries, back-flow begins through the contralateral (non-cannulated) common carotid artery. At no point do the vertebral arteries receive retrograde flow, as there is no communication between the vertebral and carotid arteries in *Odocoileus* [31]. See figures S3 and S4: Video recording of fluoroscopy-guided radiopaque latex arterial injection of *Odocoileus virginianus* in (Figure S3) lateral view, and (Figure S4) dorsal view.

Notable differences in flow patterns between *Sus* and *Odocoileus* stem from the more direct supply of blood to caudal portion of the carotid rete of *Sus*. Whereas *Sus* possesses a homologous internal carotid artery, branching directly off the common carotid, *Odocoileus* supplies the carotid rete more rostrally, via rami from the maxillary artery. Importantly, although the vascular patterns of these taxa differ, the contralateral common carotid was a consistent indicator vessel. This indicator may be broadly useful for cranial latex injections of macrovertebrates that possess paired common carotid arteries, and we advocate its use for cranial arterial injections of most mammals.

## Discussion

The aim of this study was to develop a method to reduce uncertainty in radiopaque vascular injection techniques. Vascular injection is a highly variable method–the rate and pressure at which any fluid medium is injected into the specimen depends on a number of factors, including the viscosity of the fluid, the caliber of large and distributing vessels, the number and extent of capillary beds, the size of the animal, and its state of preservation. As a result, gauging the extent of perfusion is difficult. Specimens of interest for vascular studies are frequently valuable and rare, so obtaining maximal data and avoiding destruction of specimens is of paramount importance. Although our available fluoroscopic imaging equipment is bi-planar, we propose utilization of any fluoroscopy equipment to guide the injection process because it allows real-time monitoring of perfusion. Fluoroscopy has been a useful method in vascular studies for decades. Primarily used in angiographic studies in a medical context, fluoroscopy has advanced the fields of cardiology [32], [33], oncology [34], and orthopedics [35]. Biplanar cinefluoroscopy, in specific, has increased the safety and accuracy of spinal surgeries [36] and fitting of orthopedic implants [37]. This is the first application of fluoroscopy to radiopaque latex vascular injection.

We found that monitoring perfusion in real-time using fluoroscopy to be highly effective for ensuring the success of vascular perfusion. This method enables researchers to adjust the pressure with which the radiopaque medium is injected and to terminate injection at an appropriate point. Vessels that begin to swell during injection can be immediately identified as blow-out risks, and the pressurized injection medium can be allowed to equilibrate before the injection process continues, or injection can be halted until an alternative artery is cannulated. Using these types of responses, five out of five injections performed for this study resulted in nearly ideal perfusion of cranial arteries. In addition to guiding injections, we found that fluoroscopy enables observation of flow patterns for radiopaque fluids pumped through the common carotid artery. Beyond cranial vascular studies, video fluoroscopy has been a useful technique for observation of fluid flow through many anatomical systems [38], [39]. Furthermore, this study has yielded consistent markers for complete perfusion of cranial arteries. These include perfusion of radiopaque injection medium into the rostral-most vessels (internally) and back-flow through the contralateral (non-cannulated) common carotid artery (externally). We recommend use of this external marker over other indicators for perfusion (e.g. observation of mucosal tissues).

## Supporting Information

Figure S1 **Video recording of fluoroscopy-guided radiopaque latex arterial injection of** ***Sus scrofa domestica*** **in (1) lateral view, and (2) dorsal view.** Fluoroscope settings: fluoroscope 1 (lateral) = 87 kVP and 3.8 mA; fluoroscope 2 (dorsal) = 90 kVp and 4.0mA.
(ZIP)

Figure S2 **Video recording of fluoroscopy-guided radiopaque latex arterial injection of** ***Sus scrofa domestica*** **in (1) lateral view, and (2) dorsal view.** Fluoroscope settings: fluoroscope 1 (lateral) = 87 kVP and 3.8 mA; fluoroscope 2 (dorsal) = 90 kVp and 4.0mA.
(MP4)

Figure S3 **Video recording of fluoroscopy-guided radiopaque latex arterial injection of** ***Odocoileus virginianus*** **in (1) lateral view, and (2) dorsal view.** Fluoroscope settings: fluoroscope 1 (dorsal) = 80 kVp and 3.5 mA; fluoroscope 2 (lateral) = 70 kVP and 3.5 mA.
(MP4)

Figure S4 **Video recording of fluoroscopy-guided radiopaque latex arterial injection of** ***Odocoileus virginianus*** **in (1) lateral view, and (2) dorsal view.** Fluoroscope settings: fluoroscope 1 (dorsal) = 80 kVp and 3.5 mA; fluoroscope 2 (lateral) = 70 kVP and 3.5 mA.
(ZIP)
